# Synovial mesenchymal progenitor derived aggrecan regulates cartilage homeostasis and endogenous repair capacity

**DOI:** 10.1038/s41419-022-04919-1

**Published:** 2022-05-18

**Authors:** Roman J. Krawetz, Yiru Elizabeth Wu, Karri L. Bertram, Anchita Shonak, Anand O. Masson, Guomin Ren, Catherine Leonard, Mohit Kapoor, John R. Matyas, Paul T. Salo

**Affiliations:** 1 McCaig Institute for Bone and Joint Health, University of Calgary, Calgary, AB Canada; 2 Department of Cell Biology and Anatomy, University of Calgary, Calgary, AB Canada; 3 Department of Surgery, University of Calgary, Calgary, AB Canada; 4 Biomedical Engineering Graduate Program, University of Calgary, Calgary, AB Canada; 5 Krembil Research Institute, University Health Network, and University of Toronto, Toronto, ON Canada; 6 Department of Comparative Biology and Experimental Medicine, University of Calgary, Calgary, AB Canada

**Keywords:** Mesenchymal stem cells, Cartilage, Experimental models of disease

## Abstract

Aggrecan is a critical component of the extracellular matrix of all cartilages. One of the early hallmarks of osteoarthritis (OA) is the loss of aggrecan from articular cartilage followed by degeneration of the tissue. Mesenchymal progenitor cell (MPC) populations in joints, including those in the synovium, have been hypothesized to play a role in the maintenance and/or repair of cartilage, however, the mechanism by which this may occur is unknown. In the current study, we have uncovered that aggrecan is secreted by synovial MPCs from healthy joints yet accumulates inside synovial MPCs within OA joints. Using human synovial biopsies and a rat model of OA, we established that this observation in aggrecan metabolism also occurs in vivo. Moreover, the loss of the “anti-proteinase” molecule alpha-2 macroglobulin (A2M) inhibits aggrecan secretion in OA synovial MPCs, whereas overexpressing A2M rescues the normal secretion of aggrecan. Using mice models of OA and cartilage repair, we have demonstrated that intra-articular injection of aggrecan into OA joints inhibits cartilage degeneration and stimulates cartilage repair respectively. Furthermore, when synovial MPCs overexpressing aggrecan were transplanted into injured joints, increased cartilage regeneration was observed vs. wild-type MPCs or MPCs with diminished aggrecan expression. Overall, these results suggest that aggrecan secreted from joint-associated MPCs may play a role in tissue homeostasis and repair of synovial joints.

## Introduction

Osteoarthritis (OA) is characterized by a persistent homeostatic imbalance, with catabolism outpacing anabolism, which accompanies articular cartilage degeneration, joint pain, and disability [1, 2]. A hallmark of OA is the loss of proteoglycan, a.k.a. aggrecan, which compromises the function of cartilage and further exacerbates joint damage/degeneration [3]. Aggrecan, the major proteoglycan of articular cartilage, binds noncovalently with hyaluronan (HA) to form large, hydrophilic macromolecular complexes that resist joint compression and endow cartilage with its viscoelastic material properties [4, 5]. The *ACAN* gene encodes a ~300 kDa core protein that is modified post-translationally with polyanionic chondroitin and keratan sulfate glycosaminoglycans (GAG) [6]. The aggrecan core protein is comprised of three globular regions (G1, G2, G3) separated by interglobular domains (G1–G2) and a GAG-binding region (G2–G3) [7]. The N-terminal G1 domain anchors aggrecan to HA, the C-terminal G3 domain links the proteoglycans the ECM [7]. Whereas the exact function of the G2 domain is yet established, it is thought to play a role in protein secretion [7].

Since aggrecan is necessary for cartilage function and is expressed in tissues other than cartilage at low levels [8–10], it is widely used as a marker of in vitro chondrogenesis within stem/progenitor cells (MPCs, ESCs, iPSCs) [11, 12]. Typically, an increase in aggrecan mRNA expression is observed post-chondrogenic induction, suggesting the presence of a biologically relevant ECM; yet undifferentiated bone marrow-derived MPCs also express aggrecan [13]. While bone marrow-derived MPCs are a promising source of transplantable stem cells for treating OA joints, it has been shown that the synovium contains a resident MPC population which has greater chondrogenic capacity compared to marrow- or adipose-derived MPCs [14]. Furthermore, in rodents [15, 16] and rabbits [17], synovial MPCs home to the site of cartilage injury, undergo chondrogenesis, and stimulate cartilage repair in vivo. We and others have reported a seemingly paradoxical decrease in the chondrogenic potential of synovial MPCs derived from OA joints compared to normal controls [18–21]. Yet, the overall number of synovial MPCs increases in OA joints and is positively correlated with the disease severity [22], which could be interpreted as repair response; albeit insufficient to prevent OA progression.

During the pathogenesis of OA, aggrecan metabolism itself is altered. Aggrecan core protein can be cleaved such that one fragment remains bound to HA and retained within the cartilage while the remainder becomes disaggregated [23, 24] and diffuses into the synovial fluid [23]. When proteolysis occurs within the interglobular domain or GAG-attachment region, the size/charge is disrupted, resulting in a decreased ability of cartilage to resist compression [25].

Although abundant in cartilage and responsive to mechanical loading, aggrecan is present in the cardiovascular and nervous systems [26] and during organogenesis [27], suggesting that aggrecan has yet undefined roles beyond the musculoskeletal system.

The present study explores the intriguing differential expression of aggrecan in undifferentiated MPCs in normal and OA joints to gain a more complete understanding in its role in healthy homeostasis and its dysregulation in joint disease.

## Results

### Localization of aggrecan in normal vs. OA synovial MPCs

**Fig. 1:**
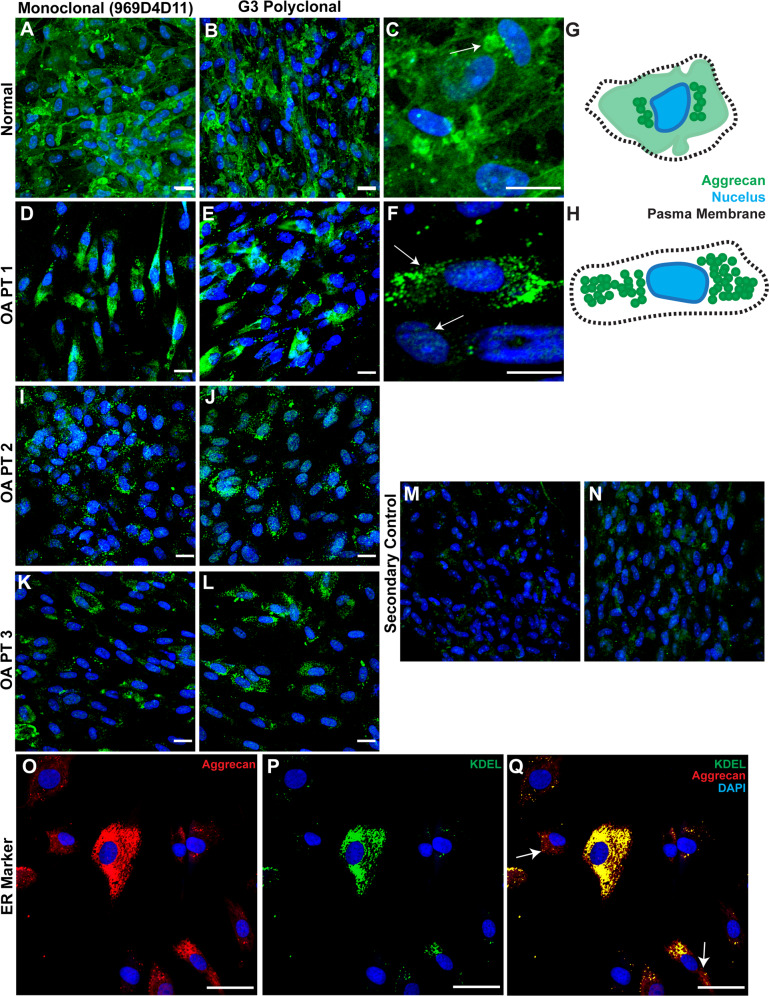
Aggrecan localization in normal and OA synovial MPCs. In normal synovial MPCs, aggrecan staining is present throughout the cytoplasm (**A**–**C**), with areas of increased staining observed adjacent to the nucleus (**C**, arrow, **G**—representation). In OA synovial MPCs, little to no cytoplasmic staining is observed (**D**–**L**), instead intense staining is observed only adjacent to the nucleus (**F**, arrows, **H**—representation). When OA MPCs were doubled labeled for aggrecan (G3 polyclonal) (**O**) and a marker of the endoplasmic reticulum (KDEL, **P**), the majority, but not all of the aggrecan staining appeared to be within the endoplasmic reticulum (**Q**, arrows). Secondary antibody alone controls demonstrate minimal non-specific staining (**M**, **N**). Scale bars equal 10 µm (**A**–**L**), 20 µm (**O**–**Q**).

The subcellular localization of aggrecan was investigated in synovial MPC lines (*n* = 8 normal, *n* = 8 OA). In synovial MPCs derived from normal individuals, aggrecan staining was diffuse through the cytoplasm (Fig. 1A, B), with areas of increased staining observed adjacent to the nucleus in a punctate pattern (Fig. 1C arrow, G). In synovial MPCs from patients with OA (Fig. 1D–L), aggrecan staining was punctate and located adjacent to the nucleus (Fig. 1F, arrows, H). Two aggrecan antibodies were used to ensure that staining patterns were consistent: one toward the G1 domain (Fig. 1A, D, I, K); and one toward the G3 domain (Fig. 1B, E, J, L). Both antibodies demonstrated consistent staining patterns in normal and OA synovial MPCs. To determine aggrecan localization in OA MSCs, cells were double labeled with aggrecan (Fig. 1O) and KDEL (endoplasmic reticulum (ER) marker, Fig. 1P). This revealed substantial co-localization of aggrecan with KDEL (Fig. 1Q), yet some aggrecan staining was distinct from KDEL (Fig. 1Q, arrows). This suggests that the majority of aggrecan is retained in the ER in OA synovial MPCs.

### Quantification of punctate aggrecan staining

**Fig. 2:**
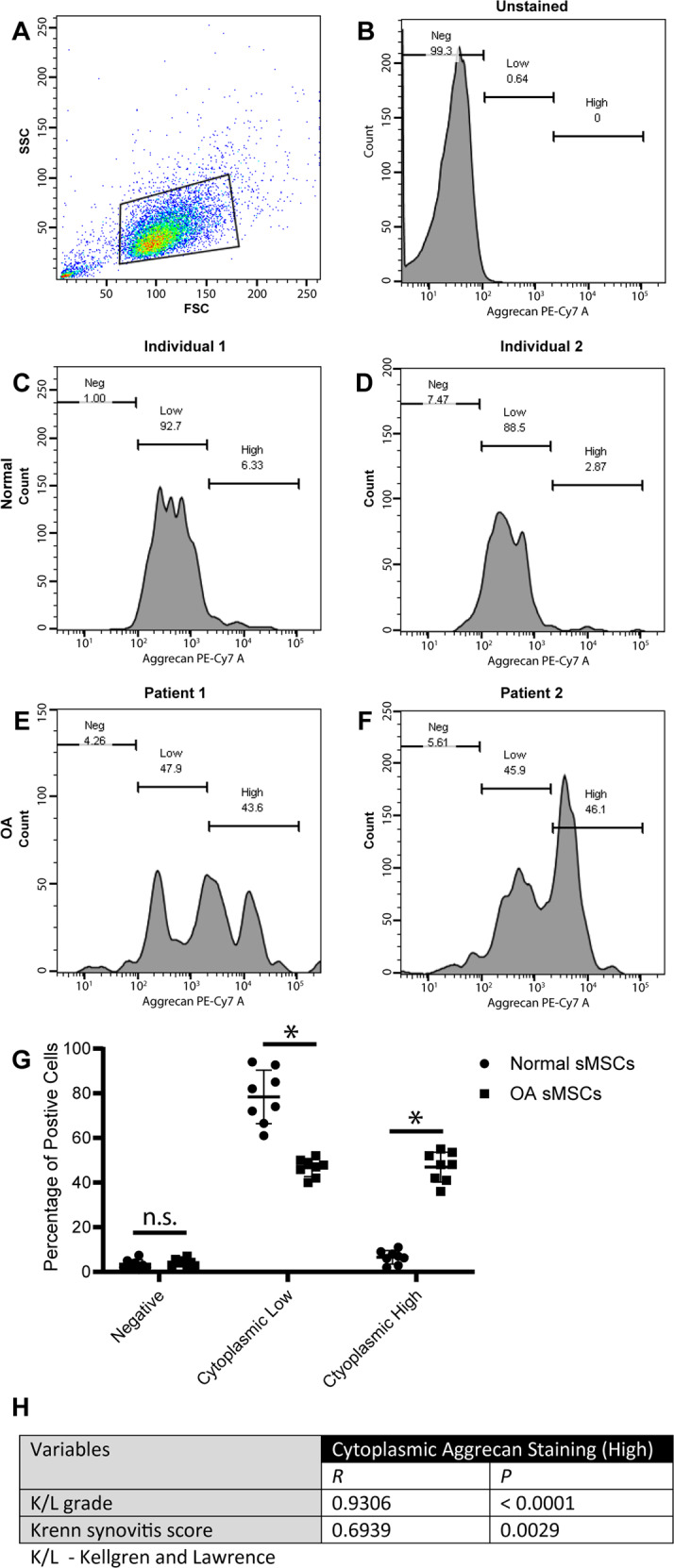
Enumeration of sub-populations of synovial MPCs with abnormal aggrecan staining. Intracellular flow cytometry was employed to quantify cells with intense aggrecan staining adjacent to the nucleus within normal vs. OA synovial MPC populations. Overall synovial MPCs from normal and OA donors maintained a similar forward and side-scatter appearance (**A**), with unstained MPCs demonstrated little to no autofluorescence in the PE-Cy7 channel (**B**). Most MPCs from two representative normal individuals were positive for aggrecan (low gate: **C**, **D**) with few cells present in the high/bright gate. However, there was an equal split between low-positive and high-positive cells in the OA MPCs tested (**E**, **F**), with almost all cells positive for aggrecan staining. When data from all normal (*n* = 8) and OA (*n* = 8) patients was quantified, ~50% of OA MPCs demonstrated this abnormal aggrecan staining, while this same staining pattern was observed in ~5% of normal MPCs (**G**). A positive correlation was observed between cytoplasmic aggrecan staining and both K/L and synovitis score (**H**). *p < 0.05.

To quantify cell populations with increased punctate aggrecan staining, an intracellular flow cytometry approach was utilized (Fig. 2). Synovial MPCs from normal and OA individuals demonstrated similar forward and side-scatter parameters (Fig. 2A) with minimal PE-Cy7 autofluorescence (Fig. 2B), hence, a gating strategy was defined as: negative (*x* < 10^2^), low-positive (*x* > 10^2^; *x* < 10^3.2^), and high-positive (*x* > 10^3.2^) aggrecan staining. In synovial MPCs from normal individuals, the majority of the cells were in the low-positive gate (Fig. 2C, D), while in the OA synovial MPCs, there was a split between low-positive and high-positive gates. When normal and OA samples were quantified (*n* = 8 each group), a decrease in low aggrecan gating with an increase in high aggrecan gated was observed in OA MPCs (Fig. 2G). It is noteworthy that nearly all synovial MPCs (in normal or OA) stained positive for aggrecan (>95% of the population). To determine if the proportion of high-positive cells changed with OA progression or synovial inflammation, a correlation analysis was undertaken. The normal group presented with K/L grades 0 and 1, and Krenn scores [28] ranging between 0–3 (mean 1.2). The OA group presented with K/L grades 3 and 4, with Krenn scores ranging between 6–10 (mean 7.9). A significant positive correlation was detected between aggrecan high-positive cells and both K/L grade and synovitis score (Fig. 2H).

### Aggrecan secretion

**Fig. 3:**
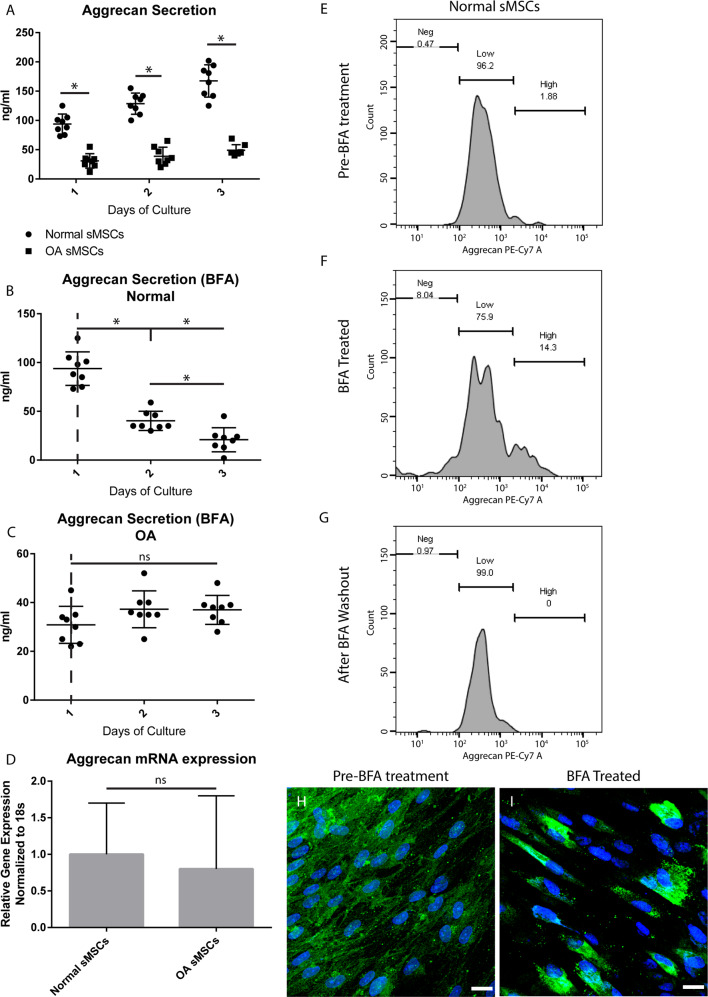
Aggrecan secretion. Aggrecan secretion from normal and OA MPCs was analyzed by ELISA and it was found that OA MPCs secreted significantly less aggrecan than normal MPCs at all time points examined (**A**). To test if this phenotype was in part due to the inability of aggrecan to leave the ER/Golgi complex, normal (**B**) or OA (**C**) MPCs were treated with Brefeldin A (BFA). BFA treatment of normal MPCs significantly decreased aggrecan expression (**B**), while BFA treatment had no effect on aggrecan secretion in OA MPCs (**C**). To control for a difference in gene expression, mRNA in normal and OA MPCs was quantified, and no difference was observed (**D**). Flow cytometry (**E**–**G**) and immunofluorescence (**H**, **I**) was used to examine if BFA treatment of normal MPCs resulted in the same aggrecan staining phenotype observed in OA cells. BFA treatment resulted in a shift to high-positive gate (**E**, **F**) that was reversible with removal of BFA (**G**) and the intense aggrecan staining adjacent to the nucleus was also observed with BFA treatment (**I**). Scale bars equal 10 µm. *p < 0.05.

We sought to determine if synovial MPCs can secrete aggrecan, and furthermore, if the high-positive phenotype observed in OA MPCs predicts the level of aggrecan secretion. Cell culture supernatant was collected from normal and OA synovial MPC lines (*n* = 8/group) and analyzed for aggrecan by ELISA (Fig. 3). While both normal and OA MPC lines secreted aggrecan (Fig. 3A), normal MPCs secreted ~3x more aggrecan. To test if this difference was due to a “blockade” in aggrecan secretion in the ER/golgi pathway, normal and OA MPCs were treated with Brefeldin A (BFA), a reversible inhibitor of protein secretion [29]. Normal MPCs exposed to BFA demonstrated a decrease in aggrecan secretion (Fig. 3B), while BFA had no effect on aggrecan secretion in OA MPCs (Fig. 3C). No differences in *Acan* mRNA levels between normal and OA MPCs was observed (Fig. 3D). Therefore, subcellular aggrecan immunolocalization was undertaken under BFA conditions to determine if blocking protein secretion in normal MPCs replicated phenotype observed in OA MPCs. Normal MPCs demonstrated diffuse cytoplasmic staining of aggrecan (Fig. 3E, H). However, after treatment with BFA for 36 h (Fig. 3F, I), a sub-set of cells moved into the high-cytoplasmic staining gate (Fig. 3E) and the staining pattern of aggrecan became punctate and perinuclear (Fig. 3I). Removing BFA resulted in MPCs reverting to the low-positive staining gate (Fig. 3G). To control for a global deficiency in secretion by OA MPCs, HA [29] was quantified and no differences were observed (Fig. S1).

### Aggrecan localization in vivo: human

**Fig. 4:**
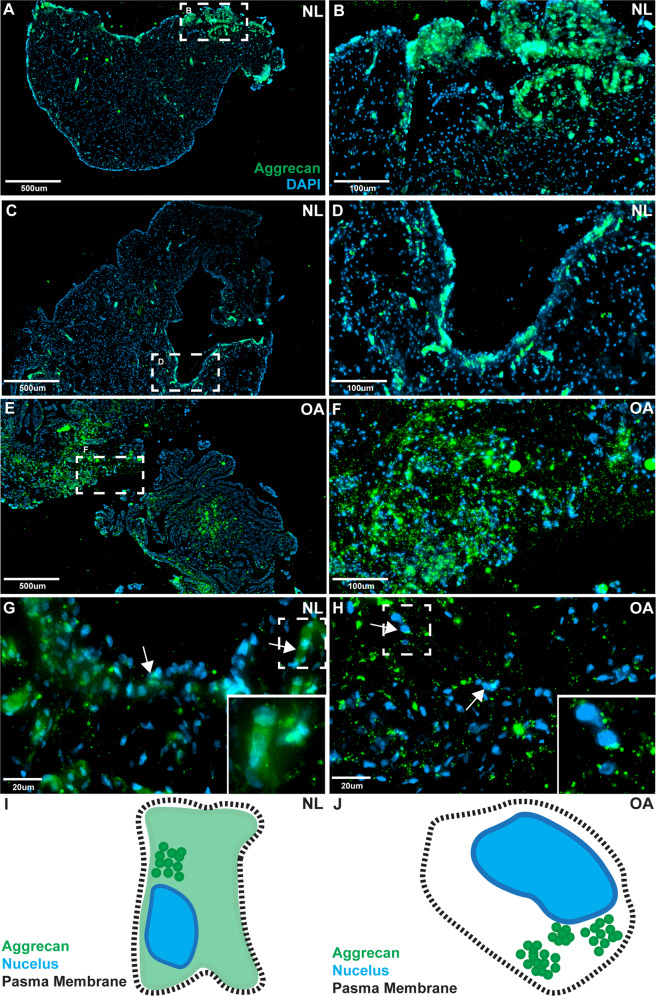
Aggrecan staining in human synovium. Normal (*n* = 8) and OA (*n* = 8) synovial biopsy samples were stained with aggrecan and DAPI. In normal (NL) synovium, aggrecan staining was predominantly observed within the intimal layer, but was also observed infrequently within the sub-intima (**A**–**D** two individuals shown as representative examples). In OA synovium, aggrecan staining was observed equally between the intima and sub-intima (**E**, **F**). The subcellular localization of aggrecan was also different between normal vs. OA samples. In normal samples aggrecan staining was diffuse throughout the cells with areas of increased staining adjacent to the nucleus (**G** arrows), while in OA samples a punctate pattern of staining was observed with increased staining adjacent to the nucleus (**H** arrows). Diagrammatic representations of aggrecan staining in normal (**I**) and OA (**J**) are also provided.

To confirm if distinct aggrecan staining patterns are present in normal and OA synovium, biopsies were immunostained for aggrecan (Fig. 4). In normal (NL) synovial samples, aggrecan staining was pronounced in the intimal layer and less intense in the sub-intimal layer (Fig. 4A, C). Higher magnification demonstrates a diffuse cytoplasmic staining similar to the pattern observed within cultured MPCs from normal synovium (Fig. 4B, D). In OA synovium, aggrecan staining was evenly distributed in the cells of the intimal and sub-intimal layers (Fig. 4E), and at higher magnification, intracellular aggrecan staining was noticeably more punctate in cells (Fig. 4F). Diffuse cytoplasmic aggrecan staining with increased staining adjacent to the nucleus was observed in normal synovium (Fig. 4G, arrows); while in OA synovium no diffuse cytoplasmic staining was observed, instead a punctate pattern was seen adjacent to DAPI nuclear staining (Fig. 4H, arrows).

### Aggrecan subcellular localization in vivo: rat

**Fig. 5:**
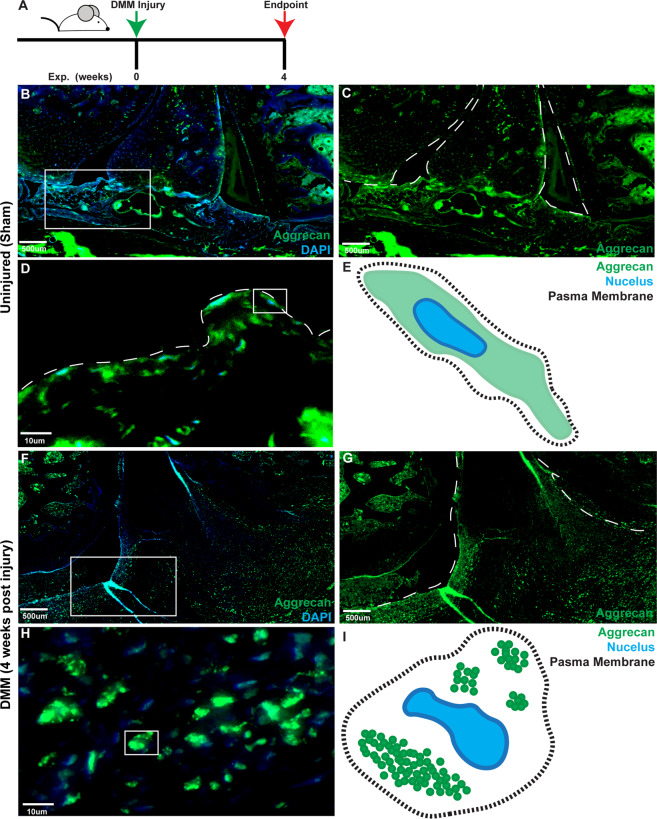
Aggrecan staining in rat synovium. Normal (*n* = 5) and DMM induced OA (*n* = 5) rat joints (**A**) were stained with aggrecan and DAPI. In normal synovium aggrecan staining was observed within the intimal and the sub-intimal (**B**, **C**) layers and was diffuse throughout the cell (**D** diagrammatic representation **E**). In OA synovium, aggrecan staining was throughout the intima and sub-intima (**F**, **G**). In OA synovium a punctate pattern of staining was observed in the synovium (**H** diagrammatic representation **I**).

To investigate if the punctate aggrecan staining pattern observed throughout human OA synovium and in MPCs from OA patients was associated with the onset/progression of OA, we examined the aggrecan staining pattern in rats post-DMM surgery (Figs. 5A and S2). In control rat joints, aggrecan can be seen within the synovium (Fig. 5B, C) and diffuse throughout cells in the intimal and sub-intimal regions (Fig. 5D, E). Post-DMM surgery, aggrecan staining is throughout the synovium (Fig. 5F, G) and, the pattern appears to be punctate (Fig. 5H, I). This punctate staining pattern was not due to cells undergoing apoptosis, as they negative for Caspase-3 (Fig. S2).

### Co-localization with MPC markers in vivo

**Fig. 6:**
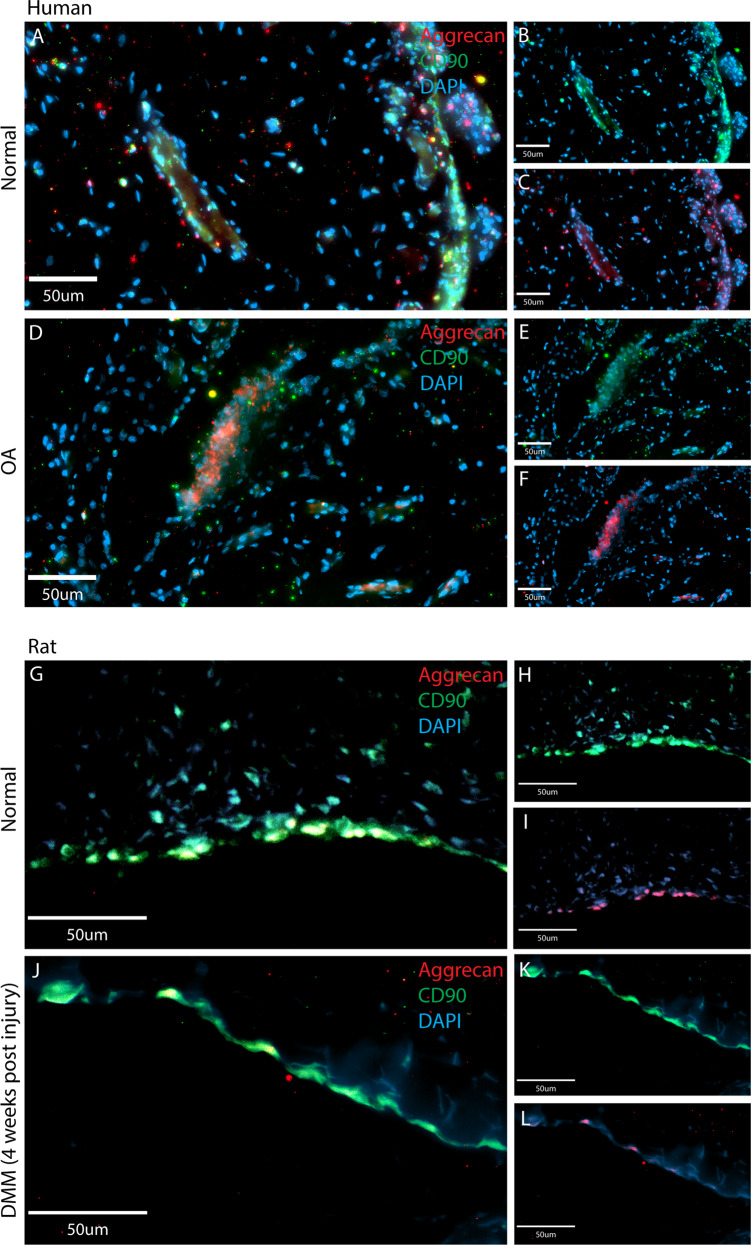
Aggrecan staining co-localizes with CD90 expressing in human and rat synovium. Human (normal and OA) and rat (normal and OA) synovium was doubled labeled for aggrecan (red) and CD90 (green), a marker of synovial progenitor/MSCs in both human and rat. In normal human synovium (**A**), CD90 (**B**) and aggrecan (**C**) were found to be highly co-localized, mainly at the intimal surface, but also within the sub-intimal layer adjacent to vessels. In OA synovium (**D**), CD90 (**E**) and aggrecan (**F**) were also found to be highly co-localized in the intimal and sub-intimal layers. In normal rat synovium (**G**), CD90 (**H**) positive cells were also found to express aggrecan (**I**). In OA synovium (**J**), CD90 (**K**) positive cells also expressed aggrecan (**L**). Scale bars equal 50 µm.

It remained undetermined which cells within the synovium were expressing aggrecan in vivo. As CD90 is a common marker of human and rat MPCs, and our synovial MPCs were purified based on their expression of CD90, sections of rat and human synovium were stained with CD90 and aggrecan (Fig. 6). In normal human synovium, CD90-positive cells were observed in the intimal layer, with straining observed around vessels (Fig. 6A, B), with aggrecan staining co-localized with CD90 (Fig. 6A, C). In OA synovium, CD90-positive cells were observed in the intimal and sub-intimal layers (Fig. 6D, E) and co-localized with aggrecan staining (Fig. 6D, F). In normal rat joints, CD90-positive cells within the synovium (Fig. 6G, H) co-localized with aggrecan (Fig. 6I). In joints post-DMM surgery, CD90-positive cells were observed within the synovium (Fig. 6J, K) and co-localized with aggrecan (Fig. 6L).

### Differential gene expression in OA MPCs

To interrogate the mechanism behind the aggrecan localization phenotype in OA MPCs, normal and OA (*n* = 3 each) MPCs underwent microarray analysis. Overall, 208 genes were found to be differentially expressed (±3fold) between normal and OA MPCs. Functional annotation was performed based on GO term enrichment to identify genes associated with mechanisms of protein secretion. While no genes were identified with ER/golgi apparatus secretory function, six genes were identified that play a role in vesicle formation and/or protein transport (Table 1). The expression levels of CD36, A2M, FIGF, PECAM1, SRGN and THBS1 were validated qPCR in normal and OA (*n* = 8 each) MPCs with only FIGF not reaching significance (Table 1). While a number of these genes have been previously associated with aggrecan and/or cartilage previously, we focused on A2M (Alpha-2-macroglobulin) as it acts as a sacrificial peptide to protect aggrecan from protease degradation [30] in addition to being a chondro-protective agent when used in a rat OA model [31].

**Table 1:** Differentially regulated genes related in protein secretion in OA vs. normal synovial MPCs.

Gene symbol	Gene name	Microarray (fold difference) OA vs. normal	qPCR (fold difference) OA vs. normal
CD36	CD36 molecule (thrombospondin receptor)	−36.54*	−56.12*
A2M	Alpha-2-macroglobulin	−10.64*	−25.54*
FIGF	c-fos induced growth factor	−4.44*	−2.12^NS^
PECAM1	Platelet/endothelial cell adhesion molecule	−3.85*	−3.11*
SRGN	Serglycin	4.84*	8.57*
THBS1	Thrombospondin 1	9.46*	17.65*

*NS* not significant.

*p < 0.05.

### Rescue of alpha-2-macroglobulin expression in OA synovial MPCs

**Fig. 7:**
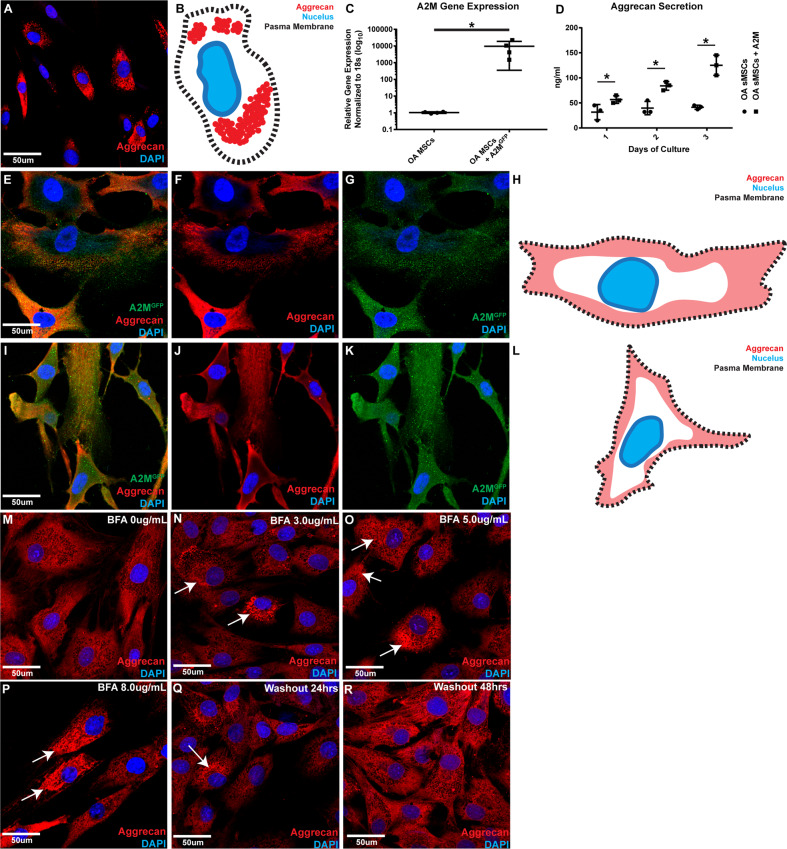
Overexpression of A2M alters aggrecan staining pattern and increase secretion in OA synovial MPCs. Un-transfected OA synovial MPCs demonstrating intense, punctate, perinuclear aggrecan staining pattern adjacent to the nucleus as observed previously (**A** diagrammatic representation **B**). OA MPCs also demonstrated low expression levels of A2M, and this could be rescued through A2M overexpression (**C**). In OA MPCs, A2M overexpression increased the secretion of aggrecan during all time-points examined when compared to the un-transfected parental cell lines (**D**). With A2M overexpression in OA MPCs (two patient cell lines shown as representative examples: **E**, **I**), aggrecan staining was no longer observed adjacent to the nucleus (**F**, **J** diagrammatic representation **H**, **L**). Instead A2M expression (GFP) was observed throughout the cytoplasm of the transfected cells (**G**, **K**). BFA treated of A2M transfected MPCs results in a redistribution of aggrecan within the cells directly related to the BFA concentration (**M**–**P**). This phenotype can be reversed by removing the BFA (**Q**, **R**). Scale bars equal 50 µm. *p < 0.05.

OA MPCs (*n* = 3) were employed for the A2M rescue experiments. Each was analyzed for aggrecan subcellular localization, A2M mRNA expression, and aggrecan secretion. A full length GFP-tagged A2M was transfected into OA MPCs, and cells were sorted based on GFP expression. In un-transfected parental cell lines, aggrecan immunostaining was punctate and adjacent to the nucleus (Fig. 7A, B). Post-A2M transfection the GFP-positive OA MPCs overexpressed A2M compared to the parental cells (Fig. 7C). While the parental cells demonstrated low levels of aggrecan secretion, A2M overexpressing MPCs demonstrated significant increases in aggrecan secretion (Fig. 7D). Furthermore, OA MPCs overexpressing A2M demonstrated little perinuclear aggrecan staining (Fig. 7E–K, 2 examples shown), instead the aggrecan staining pattern was diffuse throughout the cytoplasm (Fig. 7F, J). It should also be noted that OA MPCs overexpressing A2M were noticeably larger than parental cells, however, these changes (or changes in aggrecan behavior) were not observed in the empty vector control (Supplementary S3). To determine if A2M overexpression was related to the redistribution of aggrecan within MPCs, the transfected cells were treated with BFA (Fig. 7M–P). Interestingly, at 3 ug/ml BFA, only a small number of cells started to revert back to the abnormal aggrecan phenotype, whereas this concentration was sufficient to induce a dramatic phenotype in normal MPCs (Fig. 3H, I). At increasing concentrations of BFA (5 and 8 ug/ml) the typical OA MPC aggrecan staining was observed (Fig. 7O, P). This phenotype was reserved with the removal of BFA (Fig. 7Q, R).

As overexpression of A2M results in a clear redistribution of aggrecan, we investigated a potential role of A2M as a sacrificial peptide for aggrecan. Therefore, the microarray data was re-analyzed to explore pathways related to proteoglycan catabolism, particularly ADAMTS and MMP gene expression. Though no differences were observed in ADAMTS, MMPs 1, 3 and 10 were increased in OA MPCs, which was validated by qPCR (Table S1).

### Injection of aggrecan reduces cartilage degeneration post-injury

**Fig. 8:**
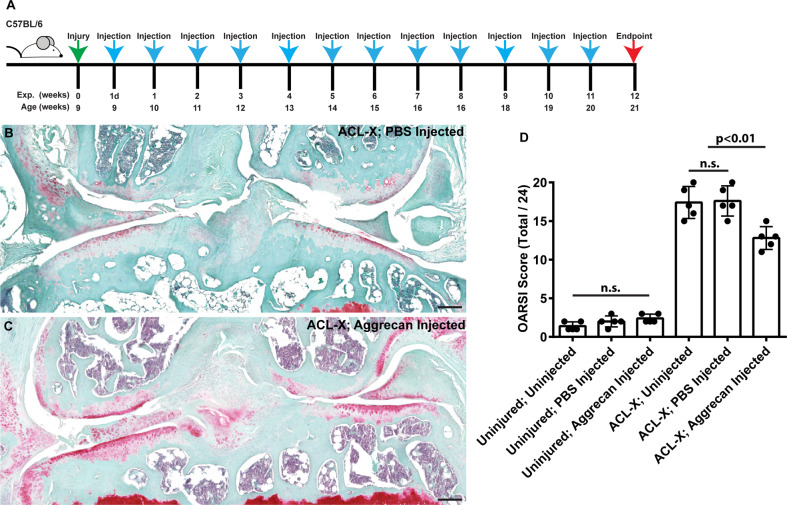
Injection of aggrecan into mice post-ACL-X in chondro-protective. Mice with or without joint injury (ACL-X) were subjected to intraarticular injection (**A**). Mouse joints without ACL-X injury, but with injection of aggrecan show little difference vs. non-injected joints in terms OARSI score (**D**). ACL-X injured joints post-aggrecan injection demonstrate an increased level of Safranin O staining in the cartilage surfaces (**B**) vs. PBS controls (**C**) and demonstrate a significant reduction in OARSI score (**D**). Scale bars equal 50 µm.

To test hypothesis that aggrecan secretion by MPCs (presumptively related to A2M-mediated aggrecan expression) contributes to joint homeostasis, we studied the treatment effect of exogenous intra-articular aggrecan on cartilage degeneration in a murine model of post-traumatic OA (ACL transection/ACL-X). Cruciate-transected mice develop significant OA pathology by 12 weeks post-injury (Fig. 8A), and the intra-articular delivery of PBS had no appreciable effect on the pathogenesis of joint degeneration (Fig. 8B, D). In contrast, intra-articular injection of aggrecan into resulted in significantly lower OARSI scores [32] (Fig. 8B, D). It is noteworthy that intra-articular injection of PBS or aggrecan into healthy, non-injured joints did not induce joint pathology (Fig. 8D).

To localize aggrecan expression in the mouse synovium, a conditional transgenic reporter (*Acan-*Tdtomato) was activated at 8 weeks of age and the joints were examined for Tdtomato expression at 9 weeks of age (Fig. S4). Tdtomato-positive cells were observed within the synovial cells in a similar distribution to both normal human and rat aggrecan staining. Furthermore, Tdtomato-positive cells were also observed near vascular structures (Fig. S4B).

### Injection of aggrecan induces cartilage repair

**Fig. 9:**
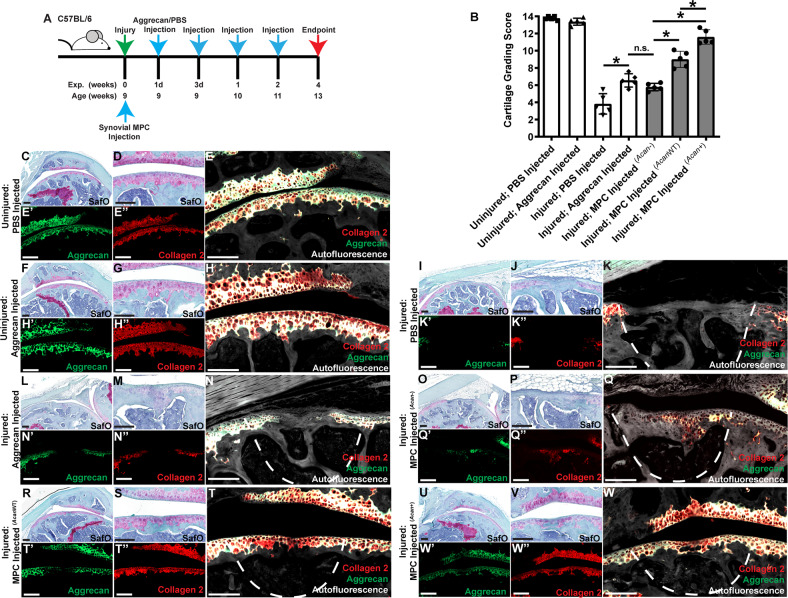
Injection of aggrecan into mice with focal cartilage defects results in increased cartilage repair. Mice underwent focal cartilage injury and intraarticular injection of aggrecan (**A**). Cartilage repair grading demonstrates an increase of cartilage repair with administration of intra-articularly delivered aggrecan (**B**). Mouse joints without injury, but with injection of aggrecan show little difference vs. non-injected joints in terms of Safranin O, collagen 2 or aggrecan staining (**C**–**H**). Injured joints post-aggrecan injection demonstrate an increased level of Safranin O, collagen 2 and aggrecan staining at the defect site (**I**–**N**). Injection of synovial MPCs from AcanCreER^T2^ (*Acan*^−^) (**O**–**Q**), C57Bl/6 (*Acan*^*WT*^) (**R**–**T**) or C57BL/6 MPCs overexpressing full length human aggrecan (*Acan*^+^) (**U**–**W**) demonstrated increasing levels of cartilage repair (**B**) and collagen 2/aggrecan staining related to their level of aggrecan expression. Scale bars equal 75 µm. *p < 0.05.

The foregoing data supports intra-articular aggrecan injection as an effective treatment for limiting joint pathology in ACL-X joints, though it is unclear whether this effect is due to slowing cartilage degeneration or whether it might promote cartilage repair/regeneration. To test the role of aggrecan on cartilage repair, we studied the effect of intra-articular aggrecan in a full-thickness cartilage defect model (FTCD) in the mouse (Fig. 9A) [16, 33]. The effects of aggrecan injection on cartilage repair was quantified using a 14 point cartilage repair scoring system [34, 35]. While complete regeneration was not achieved with this regimen of aggrecan injection, injected joints scored significantly higher compared to control (PBS)-injected joints (Fig. 9B).

In uninjured joints, we detected no striking difference in Safranin O histochemistry or collagen 2 or aggrecan immunostaining with or without intra-articular aggrecan injection (Fig. 9C–H). In joints with FTCD injury but without aggrecan, articular cartilage defects were characterized by diminished Safranin O, collagen 2, and aggrecan staining at the defect site (Fig. 9I–K). In contrast, when aggrecan was intra-articularly injected, the defect had a more uniform appearance and developed small islands of Safranin O, collagen 2, and aggrecan staining (Fig. 9L–N). While intriguing, this does not provide direct evidence regarding synovial MPC derived aggrecan, therefore, we transplanted synovial MPCs derived from AcanCreER^T2^ and C57BL/6 mice (with or without human aggrecan overexpression). AcanCreER^T2^ homozygous mice only produce ~50% of the normal amount of aggrecan protein [36], which was confirmed (Fig. S5) and therefore these mice were used as an aggrecan knock-down model. In mice receiving AcanCreER^T2^ synovial MPCs (Fig. 9O–Q), similar cartilage repair was observed to animals receiving aggrecan injections (Fig. 9B). When MPCs from C57BL/6 (Fig. 9R–T) mice or MPCs overexpressing aggrecan (Fig. 9U–W) were transplanted, an increasing degree of cartilage repair was observed, with near complete cartilage regeneration observed in mice receiving MPCs that overexpressed aggrecan (Fig. 9B).

## Discussion

While aggrecan is necessary for structure and function of cartilage [4, 5, 7], the role of aggrecan in other tissues/cells of the body is less understood. The present study describes for the first time, the presence of aggrecan within synovial MPCs. While a primary structural role for aggrecan in the synovial membrane cannot be ruled out, aggrecan was localized primarily to the cytoplasm of synoviocytes. Yet, nearly all aggrecan positive cells were also CD90-positive, but not all CD90 [37] positive cells were positive for aggrecan. This may suggest that additional cell types are being recognized by CD90 in the synovium (endothelia cells, neural cell types and potentially myofibroblasts) [38] or that not every synovial MPC expresses aggrecan in vivo. While it is known that CD90 alone is not sufficient to identify MPCs, it has been demonstrated that CD90 (in human and rat, among others) enriches for a multipotent progenitor subpopulation [37, 39] and we found that both normal and OA CD90-positive human synovial MPCs expressed aggrecan in vitro. Aggrecan expression has previously been reported in bone marrow MSCs [13], suggesting that aggrecan may play a role in MPCs within the undifferentiated state [40]. While the literature on aggrecan and MPCs focuses on aggrecan as a marker of differentiation, it is possible that aggrecan is a component of the MPC niche. While aggrecan itself has not been previously implicated in MPCs niche regulation, it is clear that ECM composition/mechanical properties play a key role in regulating the behavior of stem cells in vitro and in vivo [41]. Aggrecan produced within the synovium may serve as a reservoir for supplying other joint tissues. Since CD90-positive cells represent a minor fraction of total cells within the synovium (~2–3% of the total cell population) [42, 43] and aggrecan is a secreted protein, it is possible that MPCs secrete aggrecan into extracellular space/synovial fluid coat/infuse articular surfaces. Alternatively, resident synovial aggrecan may act locally to regulate the synthesis and localization of HA, lubricin/PRG4, and other extracellular molecules. In this study we have demonstrated that synovial MPCs (normal and OA) produce HA and it is known that these cells also produce PRG4 [19, 44, 45]. An established role of aggrecan complex with HA to increase the viscosity of synovial fluid as well as localize HA to areas of aggrecan expression (e.g., cartilage surface) [46, 47]. HA is also known to interact with PRG4 [48, 49] and this interaction regulates synovial fluid lubricating ability [50]. Therefore, it is possible that aggrecan, HA and PRG4 from synovial MPCs is secreted into the synovial fluid and that the loss of aggrecan from OA synovial MPCs might be a factor in the loss of synovial fluid viscosity/lubricating ability observed in a sub-set of OA patients [51].

In this study, while we were able to demonstrate that intra-articular injections of aggrecan are beneficial in terms of reducing cartilage degeneration and inducing cartilage repair post-injury, these experiments do not prove that aggrecan secreted from the synovial MPCs would have the same effect. Therefore, we transplanted murine synovial MPCs with varying degrees of aggrecan expression and observed a direct association between aggrecan expression in these cells with degrees of cartilage repair/regeneration.

We also decided to focus on the mechanism by which aggrecan secretion was blocked in OA MPCs. Microarray analysis was undertaken which identified a number of differentially regulated genes between normal and OA MPCs and A2M was selected for further study based on its dramatic downregulation in OA synovial MPCs; its chondro-protective effects in a rodent model of OA [31]; and its putative role as a sacrificial target for proteases that cleave aggrecan [30]. While we did not observe any differences in the gene expression of aggrecanases/ADAMTS, which would signal a potential increase in aggrecan degradation, we did observe an increased expression of a number of MMPs (MMPs 1, 3, 10) in MPCs from OA synovium. Although MMPs typically demonstrate less efficiency (vs. aggrecanases) at cleaving aggrecan [52], MMP 1 and MMP 3 can cleave aggrecan [52].

Even though we employed two aggrecan antibodies (N and C-terminal) that co-localize in both normal and OA MPCs, this approach does not conclusively demonstrate that aggrecan cleavage has not taken place. Furthermore, it has been previously shown that aggrecan cleavage in the G3 domain can lead to an accumulation of aggrecan within the ER [53]. Antibodies to these MMP-specific neo-peptides of aggrecan would be required to further test this possibility. Interestingly, A2M clinical treatment is now widely offered to patients suffering from arthritis and/or degenerative disc disease, however, little clinical evidence exists supporting the efficacy of this treatment at this time. While A2M overexpression is OA synovial MPCs promoted the release of aggrecan from the ER and increased secretion of aggrecan, it was found that the morphology of the cells was dramatically altered from the parental cell lines. Specifically, the MPCs took on a more hypertrophic appearance (flat, increased cell size). This is of interest since normal MPCs do not display this hypertrophic appearance while expressing wild-type levels of A2M (vs. OA MPCs), but also since it has been previously demonstrated that A2M treatment can induce hypertrophy in the heart [54]. This suggests that while A2M may rescue the abnormal aggrecan phenotype in OA MPCs, it may also induce side effects on cell behavior. This is important to consider as MPCs undergoing hypertrophy have severely reduced differentiation potential [55].

In conclusion, we have demonstrated that synovial MPCs express aggrecan in vitro and in vivo and that secretion of aggrecan is impaired in OA MPCs. This is directly related to a decrease in A2M expression in OA MPCs which can be rescued by overexpression of A2M. Lastly, we demonstrated that intra-articular injection of aggrecan into mouse joints inhibits cartilage degeneration in an OA model, while also inducing cartilage repair in a focal injury model. Together, these results show that aggrecan is playing a role in the joint over and above its well characterized structural roles and further research is required to determine how aggrecan produced in synovial MPCs regulates the homeostasis and/or repair response of articular cartilage.

## Materials and methods

### Subjects

A total of 16 human subjects were included in the current study. Synovial biopsies were harvested from normal (*n* = 8: 5 M, 3 F) and osteoarthritic (*n* = 8: 4 M, 4 F) knees. Normal subjects ranged in age from 32–80 years; OA subjects had an age range of 51–82.

Normal control tissue samples were obtained from the Southern Alberta Tissue Donation Program. Criteria for control cadaveric donations were an age of 30 years or older, no history of arthritis, joint injury or surgery (including visual inspection of the cartilage surfaces during recovery), no prescription anti-inflammatory medications, no co-morbidities (such as diabetes/cancer), and availability within 4 h of death. A minimum of four synovial biopsies (total) were taken by the recovery team from the medial and lateral compartments of the joint adjacent to the capsule, and care was taken to avoid the fat pad.

The inclusion criteria for OA was meeting the ACR criteria with a K/L grade of 3 or 4. Based on our ethics approval at the University of Calgary, a maximum of four synovial biopsies were obtained from OA patients and these were collected from the medial and lateral compartments of the joint adjacent to the capsule.

### Synovial MPC culture

#### Human

An outgrowth method was utilized to culture synovial MPCs. Upon receipt of synovium, three (~3 mm^3^) tissue explants were cut from the biopsy and seeded in a 24-well culture plate and incubated at 37 °C and 5% CO_2_ with 1 ml of MesenCult™ (Stemcell Technologies) culture media added. Within 11 days post-seeding, outgrown cells were adherent to the plastic and reached 30–40% confluency (referring to rough percentage of flask surface area covered with cells). At this point, cells were gently dissociated via mechanical stimulation and placed with 5 ml of MesenCult™ media in a T-25 cell culture flask. Media was changed every 3 days. After cells reached 70% confluency, the cells were washed, resuspended, and subjected to magnetic MACs purification entailing (1) hematopoietic lineage depletion (FCGR3A, CD19, CD3E, NCAM1, CD14, GYPA, FCGR3B, ITGA2B) and (2) CD90+ positive selection. Purified cells were then placed in 10 ml of MPC media in T-75 flasks. Media was changed every 3 days and cells were passaged when 70–80% confluency was reached. Cells passaged a maximum of three times were used for flow cytometry (including conformation of MPC surface marker expression), multipotent differentiation analysis and immunofluorescence. For Brefrelin A (BFA) treatment of synovial MPCs, a stock solution of 1000X (Thermo Fisher) was diluted in media to a final concentration of 3.0 ug/ml. To examine the reversible effects of the BFA treatment, the BFA containing media was removed and the cells were washed twice with DPBS before fresh media was added.

#### Mouse

Two mouse lines were used for synovial MPC harvest. C57BL6 and AcanCreER^T2^ mice (Jax labs # 019148). Homozygous AcanCreER^T2^ were employed as they are known to express ~50% of normal aggrecan expression (at the mRNA and protein levels) compared to C57BL/6 mice [36, 56]. At necropsy, the joint space was exposed under a dissecting microscope. Synovial tissue was dissected out from the joint and placed in a petri dish containing PBS and 1% anti-anti (Life Technologies). Grooves/scratches were made in the bottom of a 12 well plate with a scalpel to improve tissue transfer efficiency and to promote tissue adherence. Tissue samples were transferred to the 12 well plate with media consisting of DMEM/F-12, 10% fetal bovine serum (1% non-essential amino acid, 1% anti-anti (all Thermo Fisher). After cells reached 70% confluency, the cells were washed, resuspended, and subjected to magnetic MACs purification entailing (1) hematopoietic lineage depletion (MagniSort™ Mouse Hematopoietic Lineage Depletion Kit) and (2) Sca1+ positive selection. Cells passaged once, then underwent flow cytometry (including conformation of MPC surface marker expression).

### qRT-PCR

mRNA was collected from cultured cells using Trizol reagent (Invitrogen) and converted to cDNA using the High Capacity cDNA kit (Applied Biosystems, Carlsbad, CA). The cDNA was probed using pre-validated Taqman primer-sets for human Aggrecan (*acan*), *cd36*, *a2m*, *figf*, *pecam1, srgn, thbs1, mmp1, mmp3 and mmp10*. Human 18S rRNA was used as the housekeeping gene in all qRT-PCR experiments based on previously validated protocols.

### Aggrecan antibodies

Two aggrecan antibodies were used in this study (both Thermo Fisher): a monoclonal antibody generated against native aggrecan purified from human articular cartilage (969D4D11, Cat# AHP0022), and a rabbit polyclonal antibody for the G3 domain directed toward the sequence CDGHPMQFENWRPNQPDN (A.A. 2277-2293, Cat# PA1-1745) of human aggrecan.

### Immunofluorescence

Synovial MPCs were washed in PBS and fixed in 4% para-formaldehyde in PBS. Cells were then permeabilized in 0.5% saponin (Sigma) in PBS, rinsed once in PBS, then blocked in 3% BSA. Primary antibodies (Aggrecan, KDEL (Cat# PA1-013); all Thermo Fisher) were diluted 1:50 in 3% BSA, added to the cell samples and incubated. The cells were then washed three times with PBS and blocked again. Following the block, the cells were incubated with an appropriate Alexa Fluor 488 secondary antibody (Molecular Probes, Carlsbad, CA) and Toto-3 (Molecular Probes). After incubation, the cells were washed thrice with PBS and mounted on glass slides (9:1 glycerol:PBS). Slides were analyzed using a Zeiss 510 confocal Microscope with 488, 568 and 633 nm filters. Images were prepared using Zeiss LSM image browsing software.

### Flow cytometry

Synovial MPCs were fixed in methanol for 10 min on ice. After PBS washing, cells were permeabilized with 0.01% Triton X-100, washed in PBS and then blocked for 30 min at 37 °C with 3% BSA. They were then incubated away from light for 1 h with a fluorescent antibodies for aggrecan that directly conjugated to PE-Cy7 (Abcam) prior to flow cytometric analysis on an Invitrogen Attune® Acoustic Focusing Cytometer.

### ELISA

Aggrecan was measured in cell culture supernatant using the Aggrecan Human ELISA Kit (Thermo Fisher, Cat# KAP1461) according to the manufacturer’s protocol. HA was also measured in cell culture supernatant using the Hyaluronan DuoSet ELISA (R&D Systems, Cat# DY361405) according to the manufacturer’s protocol.

### Rat destabilization of the medial meniscus (DMM) injury model

Standardized joint injuries (*n* = 5) were created while each rat was anesthetized using isoflurane delivered in oxygen [57, 58]. A medial para-patellar arthrotomy was performed under a dissection microscope. The medial margin of the quadriceps was separated from the muscles of the medial compartment and the patella was dislocated laterally. The fat pad over the cranial horn of the medial meniscus was retracted. Sectioning of the medial meniscotibial ligament leads to a destabilization of the medial meniscus. Sham control groups did undergo surgery, but with no injury induced (*n* = 5). The joint capsule was closed with a silk suture. The skin was closed by the application of tissue adhesive. Surgery was undertaken at 12 weeks of age and 3 M and 2 F were used in each group.

### Histological analysis

Human synovial or whole rat and mouse joint sections (7 μm) were deparaffinized in CitriSolv (Fisher Scientific; Fairlawn, NJ, USA) and rehydrated through a series of graded ethanol to distilled water steps. Antigen retrieval (10 mM sodium citrate, pH 6.0, Sigma-Aldrich; St. Louis, Missouri, USA) and blocking (1:500 dilution; 100 μl goat serum: 50 ml TRIS-buffered saline, 0.1% Tween 20 (TBST) for 1 h), steps were performed prior to going through sequential wash (TBST) and the application of primary antibody. Primary antibodies conjugated to fluorophores included: Aggrecan (conjugated to AF 488 or 568), Human/Rat CD90 (BD Biosciences, (Cat# 550402)) or Caspase-3 (R&D, Cat# AF835). All slides were counterstained with the nucleic acid stain DAPI (4’,6-diamidino-2-phenylindole) (Sigma-Aldrich; St. Louis, Missouri, USA) and mounted using FluorSave reagent (Calbiochem; Darmstadt, Germany). Isotype controls for AF 488 or AF 568 demonstrated little to no reactivity. Slides were imaged using a Plan-Apochromat objective (×20/0.8 M27) on an Axio Scan.Z1 Slide Scanner microscope (Carl Zeiss; Oberkochen, Germany); DAPI (excitation 353 nm, emission 465 nm), Alexa488 (excitation 493 nm, emission 517 nm), and Alexa568 (excitation 565 nm, emission 576 nm).

### Microarray analysis

RNA was extracted using Trizol Reagent (Life Technologies, Inc, New York, USA) according to the manufacturer’s protocol. Total RNA was purified with RNeasy Plus Micro Kit (Qiagen, Valencia, USA) to remove genomic DNA. The RNA integrity number (RIN) was measured with Agilent RNA 6000 NanoChips on a 2100 Bioanalyzer (Agilent Technologies, Santa Clara, USA). The quantity was measured with a NanoDrop 1000 (NanoDrop Technologies, Inc, Wilmington, USA). A total of 300 ng of each RNA sample with RIN higher than 9 was labeled with GeneChip Whole Transcript (WT) Sense Target Labeling Assay (Affymetrix, Santa Clara, USA) and hybridized to Affymetrix GeneChip Human Gene 1.0 ST Arrays at 45 °C for 16 h. Arrays were stained and washed on Affymetrix GeneChip Fluidics 450 following the manufacturer’s protocol and scanned with an Affymetrix GeneChip Scanner 3000 7G System. Array data files were generated with GeneChip® Command Console® Software (AGCC) (Affymetrix, Santa Clara, USA) (ArrayExpress accession ID# E-MTAB-11492) and statistical analysis was carried out on software of GeneSpring^TM^ (Agilent Technologies). The fold change between normal and OA samples was based on the *p* < 0.05 from a *t*-test (Asymptotic and Benjamini Hochberg FDR).

### Overexpression of alpha-2 macroglobulin (A2M) and aggrecan

Full length human A2M or aggrecan (Origene) was sub-cloned into pcDNA3.1(+)IRES GFP (A2M) or pcDNA3.1(+) (aggrecan) (both Addgene) then transfected into OA MSCs or murine synovial MPCs respectively then selected with G418 (Thermo Fisher) for 1 week. The resultant A2M overexpressing cells were sorted based on GFP-positive signal normalized to a non-transfected parental population using a Sony SH800 cell sorter (Sony Biotechnology).

### Cartilage injury

Standardized cartilage injuries were created with the C57BL/6 mice (*n* = 5/treatment group) under isoflurane anesthesia [16, 33, 59]. A small medial para-patellar arthrotomy was performed with FTCD created using a custom-made device in which an epoxy bead was attached 200 μm from the tip of a 26G needle to control the depth of the injury. The tip of the needle was used to gently displace the patella laterally, placed in the femoral groove anterior to the intercondylar notch and rotated to make a circular full-thickness wound ~0.6 mm in diameter that penetrated into the underlying subchondral bone [33]. The patellar dislocation was then reduced and the skin closed with a wound clip. Sham control groups did undergo surgery, but with no cartilage injury induced (*n* = 5/treatment group). Surgery was undertaken at 8 weeks of age and 3 F and 2 M were used in each group.

### Anterior cruciate ligament transection (ACL-X)

Standardized injuries were created while the C57BL/6 mice (*n* = 5/treatment group) were anesthetized by isoflurane [59, 60]. A medial para-patellar arthrotomy was performed using a microsurgical scalpel. The ACL was transected with micro-scissors. Sham control groups did undergo surgery, but with no transection (*n* = 5/treatment group). The joint capsule was closed with a silk suture. The skin was closed by the application of tissue adhesive. Surgery was undertaken at 8 weeks of age and 5 F were used in each group.

### Intra-articular injection of aggrecan

Aggrecan isolated from bovine articular cartilage (Sigma) was resuspended in DPBS at a concentration of 2 mg/ml. Using a micromanipulator, a 30G needle was inserted through the tendon and into the space between the patella and the femur [16]. Entry into the joint space was considered successful when the patella could be seen lifting upwards as the needle slid underneath. A total of 2 ul of aggrecan solution was injected once per week for 4 weeks (for full-thickness cartilage injury model) or once a week for 12 weeks (for ACL-X model).

### Intra-articular injection of synovial MPCs

Intra-articular injections of Sca1+ synovial MPCs from either C57BL6 or AcanCreER^T2^ mice were performed into the knees of male C57BL6 mice aged 8 weeks as done previously [16]. With the mouse in a supine position, the knee was placed into a holder such that the fixation screws were aligned just above the patella tendon. A small skin incision was made to expose the patella and the tendon. Using the micromanipulator, a 30G needle was inserted through the tendon and into the space between the patella and the femur. Entry into the joint space was considered successful when the patella could be seen lifting upwards as the needle slid underneath. The total injection was 2 ul containing 10,000 cells. Incisions were closed using wound clips.

### Cartilage repair/OA grading

Cartilage repair in all mouse groups was evaluated after 4 weeks by scoring the full-thickness defect for cell morphology (0–4), matrix staining (0–3), surface regularity (0–3), thickness of cartilage (0–2) and integration with native cartilage (0–2) [33, 35]. On this scale, a newly created FTCD has an overall score of 0 and the (maximal) overall score for uninjured native cartilage is 14.

Mice that underwent ACL-X, were sacrificed at 12 weeks post-surgery and the joints were processed similarly to the FTCD, with the exception that frontal sections were stained. The joint was graded for OA using the OARSI Guidelines [32].

### Aggrecan lineage tracking

AcanCreER^T2^ mice (Jax labs # 019148) were bred to ROSA^TdTomato^ (Jax labs # 007909) reporter mice. At 2-months of age, the offspring received tamoxifen (4-hydroxytamoxifen, Sigma) injections for 5 consecutive days (1 mg/day) inducing Cre-mediated TdTomato labeling of *Acan-*expressing cells. Knee joints were harvested for histological analysis 1 week after the last injection.

### Data analysis and statistical procedures

Each experiment was repeated at least three times independently. Quantitative data were expressed as mean ± SD. Statistical analysis was performed with an unpaired two-tailed Student’s *t*-test or one-way Anova, and effects were considered statistically significant at *p < 0.05.

## Supplementary information

Reproducibility checklist

Supplemental Data

## Data Availability

All data are available in the main text or the Supplementary Materials.
