# Supplementary material for: Synovial mesenchymal progenitor derived aggrecan regulates cartilage homeostasis and endogenous repair capacity
Source: Cell Death Dis. 2022 May 18;13(5):470. doi: 10.1038/s41419-022-04919-1 (PMC9117284; doi:10.1038/s41419-022-04919-1)
Supplement: Supplementary file 2 — Supplemental Data [file 41419_2022_4919_MOESM2_ESM.docx]

**Supplementary Figures**

**Supplementary Figure 1. HA secretion in normal and OA synovial MPCs**. HA secretion into the culture media of normal and OA MSCs was quantified by ELISA. No difference was observed in the level of HA secretion between normal and OA MPCs.


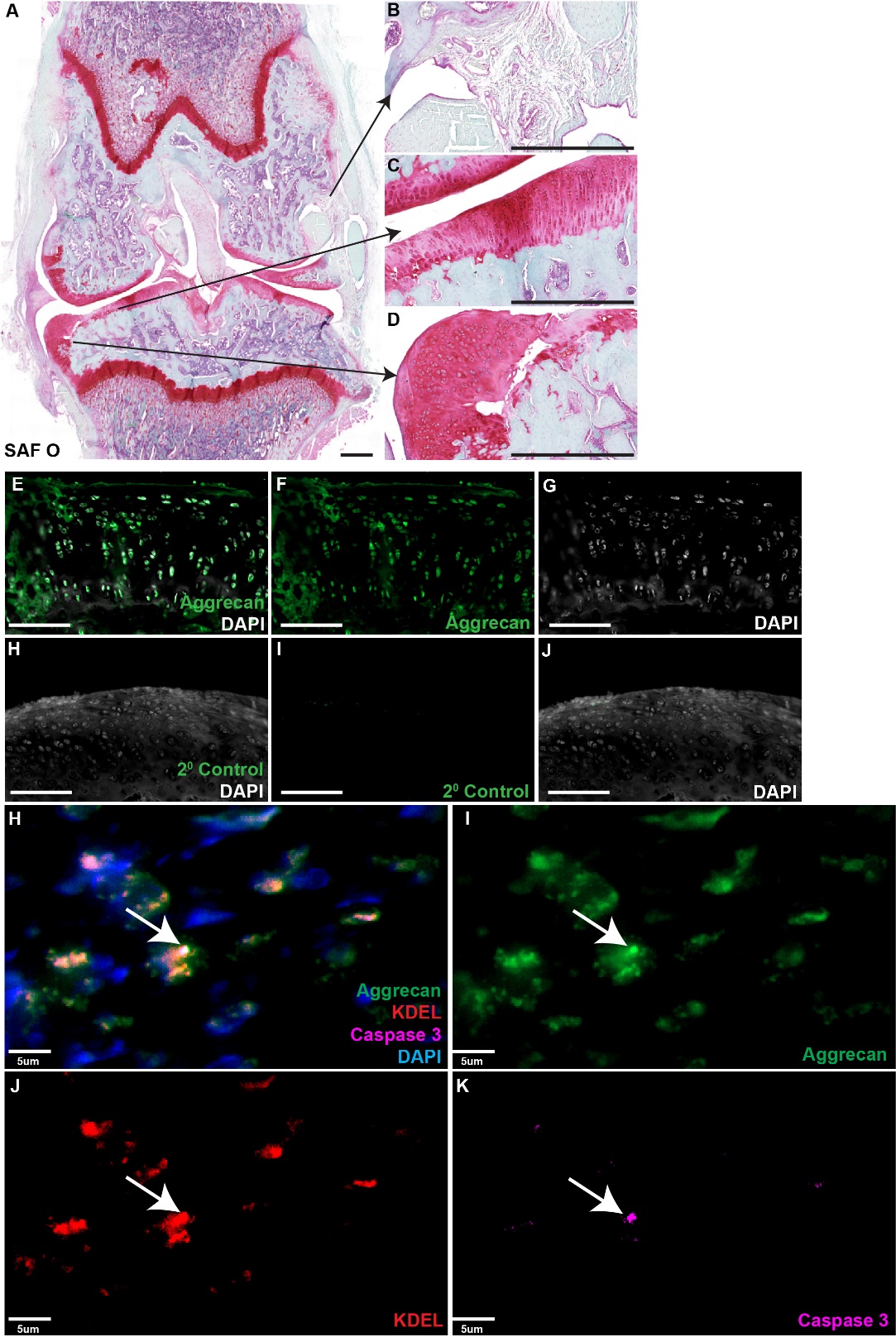


**Supplementary Figure 2. Rat DMM.** Safranin O staining of the rat joints 4 week post-DMM display changes associated with OA (A) including: synovitis (B), cartilage fibrillation/proteoglycan loss (C) and osteophyte formation (D). Scale bar for A-D equals 75µm. Aggrecan staining of rat cartilage DMM demonstrates enriched cellular expression with minimal ECM staining (E-G). 2^0^ antibody controls present with minimal background staining (H-I). Scale bar for E-J equals 50µm. Aggrecan expression within the rat OA synovium (I) co-localizes with the ER marker KDEL (J), but not with the apoptotic marker Caspase 3 (K).


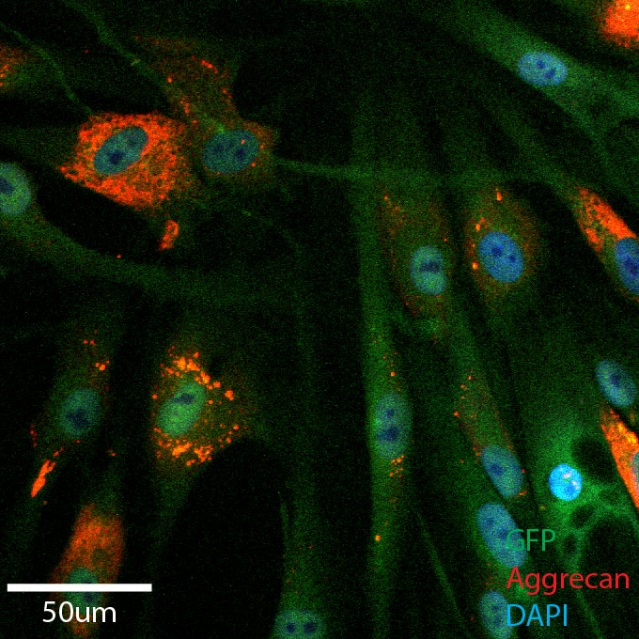


**Supplementary Figure 3. Empty vector transfection of OA synovial MPCs**. OA MPCs were transfected with the empty (no A2M ORF) vector and selected for GFP expression. Empty vector transfection did not change the localization of aggrecan in the cells, and had no effect on cell morphology.

**
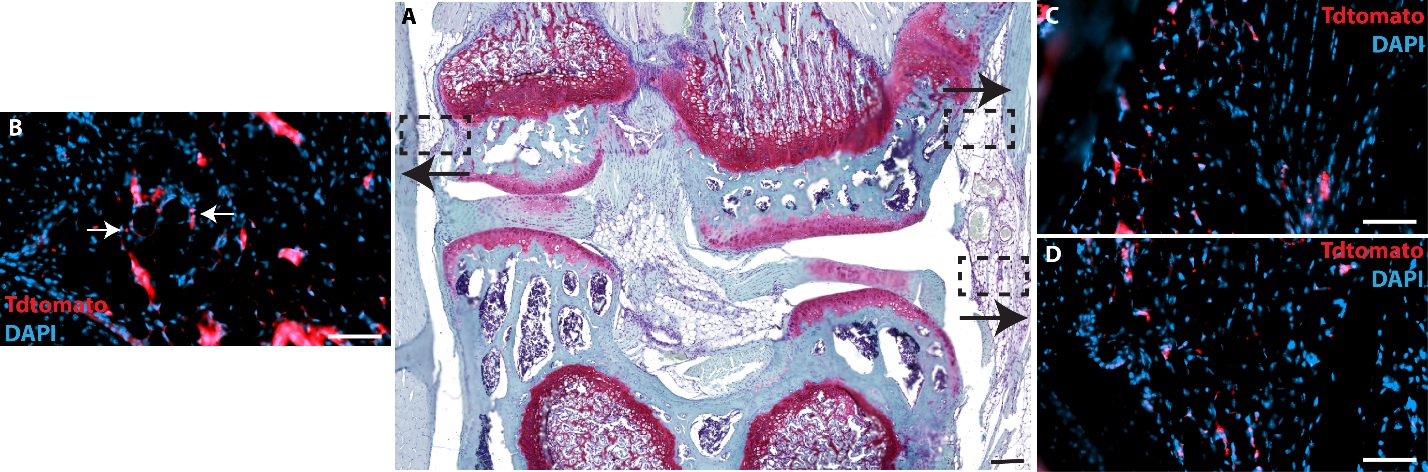
**

**Supplementary Figure 4. *Acan* expressing cells in normal mouse synovium.** *Acan* labelled cells (Tdtomato) can be observed throughout the mouse synovium (A-D). Tdtomato positive cells can also be observed associated with the vasculature (B, arrows). Scale bar equals 50µm.

**
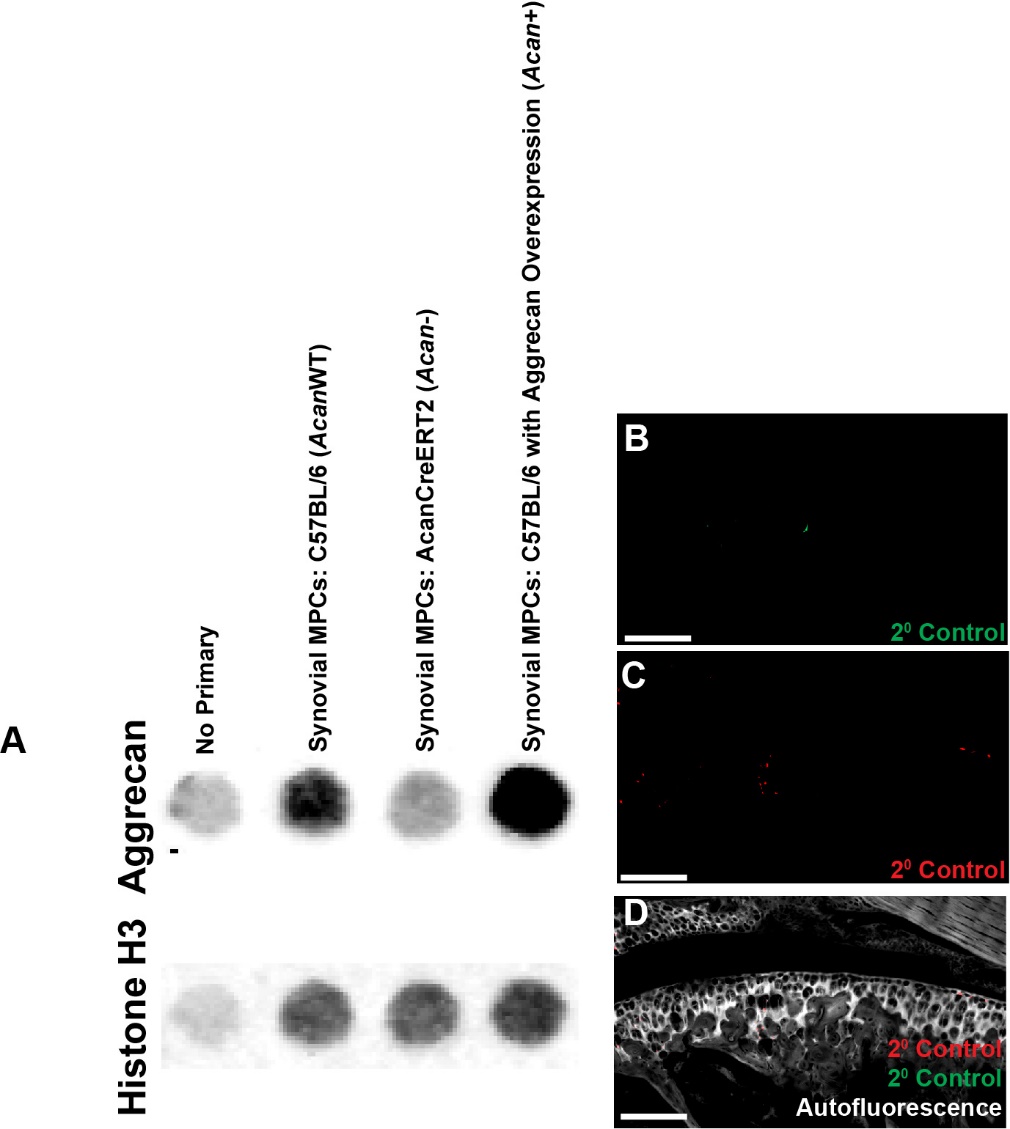
**

**Supplementary Figure 5. Aggrecan expression in synovial MPCs.** Synovial MPCs from AcanCreER^T2^ (*Acan*^-^), C57Bl/6 (*Acan^WT^*) or C57BL/6 MPCs overexpressing full length human aggrecan (*Acan*^+^) had their aggrecan protein level assayed through dot-blot with Histone H3 acting as a loading control (A). Secondary antibody controls show limited background staining (B-D). Scale bar equals 50µm.

**Supplementary Table 1. Protease gene expression in normal vs. OA synovial MPCs**. MMP1, 3, and 10 were found to be upregulated in OA MSCs by microarray and this was confirmed by qPCR. * p<0.05.

| Gene Symbol | Gene Name | Microarray  (Fold Difference)  OA vs. Normal | qPCR  (Fold Difference)  OA vs. Normal |
| --- | --- | --- | --- |
| MMP1 | Matrix metallopeptidase 1 (interstitial collagenase) | 32.79* | 28.54* |
| MMP3 | Matrix metallopeptidase 3 (stromelysin 1, progelatinase) | 17.08* | 21.36* |
| MMP10 | Matrix metallopeptidase 10 (stromelysin 2) | 4.33* | 5.91* |
